# Infrared‐Emitting Multimodal Nanostructures for Controlled In Vivo Magnetic Hyperthermia

**DOI:** 10.1002/adma.202100077

**Published:** 2021-06-12

**Authors:** Erving Ximendes, Riccardo Marin, Yingli Shen, Diego Ruiz, Diego Gómez‐Cerezo, Paloma Rodríguez‐Sevilla, Jose Lifante, Perla X. Viveros‐Méndez, Francisco Gámez, David García‐Soriano, Gorka Salas, Carmen Zalbidea, Ana Espinosa, Antonio Benayas, Nuria García‐Carrillo, Lorena Cussó, Manuel Desco, Francisco J. Teran, Beatriz H. Juárez, Daniel Jaque

**Affiliations:** ^1^ Nanomaterials for Bioimaging Group (nanoBIG) Universidad Autónoma de Madrid Madrid 28049 Spain; ^2^ IRYCIS Ctra. Colmenar km. 9.100 Madrid 28034 Spain; ^3^ IMDEA Nanociencia Faraday 9 Cantoblanco Madrid 28049 Spain; ^4^ Universidad Autónoma de Zacatecas Unidad Académica de Ciencia y Tecnología de la Luz y la Materia Carretera Zacatecas‐Guadalajara km. 6 Ejido la escondida Zacatecas Zacatecas 98160 México; ^5^ Department of Applied Physical Chemistry Universidad Autónoma de Madrid Francisco Tomás y Valiente, 7 Cantoblanco Madrid 28049 Spain; ^6^ Nanobiotecnología (IMDEA‐Nanociencia) Unidad Asociada al Centro Nacional de Biotecnología (CSIC) Madrid 28049 Spain; ^7^ Laboratory Animal Service University of Murcia Murcia 30100 Spain; ^8^ Departamento de Bioingeniería e Ingeniería Aeroespacial Universidad Carlos III de Madrid Madrid 28911 Spain; ^9^ Instituto de Investigación Sanitaria Gregorio Marañón Madrid 28007 Spain; ^10^ Unidad de Imagen Avanzada Centro Nacional de Investigaciones Cardiovasculares (CNIC) Madrid 28029 Spain; ^11^ Centro de Investigación Biomédica en Red de Salud Mental (CIBERSAM) Madrid 28029 Spain; ^12^ Present address: Madrid Institute of Materials Science (ICMM) CSIC. Sor Juana Inés de la Cruz Madrid Cantoblanco 28049 Spain; ^13^ Present address: Department of Physical Chemistry, Faculty of Science University of Granada Avenida de la Fuente Nueva S/N Granada 18071 Spain

**Keywords:** in vivo imaging, luminescence thermometry, magnetic hyperthermia, near‐infrared fluorescence, silver sulfide nanoparticles

## Abstract

Deliberate and local increase of the temperature within solid tumors represents an effective therapeutic approach. Thermal therapies embrace this concept leveraging the capability of some species to convert the absorbed energy into heat. To that end, magnetic hyperthermia (MHT) uses magnetic nanoparticles (MNPs) that can effectively dissipate the energy absorbed under alternating magnetic fields. However, MNPs fail to provide real‐time thermal feedback with the risk of unwanted overheating and impeding on‐the‐fly adjustment of the therapeutic parameters. Localization of MNPs within a tissue in an accurate, rapid, and cost‐effective way represents another challenge for increasing the efficacy of MHT. In this work, MNPs are combined with state‐of‐the‐art infrared luminescent nanothermometers (LNTh; Ag_2_S nanoparticles) in a nanocapsule that simultaneously overcomes these limitations. The novel optomagnetic nanocapsule acts as multimodal contrast agents for different imaging techniques (magnetic resonance, photoacoustic and near‐infrared fluorescence imaging, optical and X‐ray computed tomography). Most crucially, these nanocapsules provide accurate (0.2 °C resolution) and real‐time subcutaneous thermal feedback during in vivo MHT, also enabling the attainment of thermal maps of the area of interest. These findings are a milestone on the road toward controlled magnetothermal therapies with minimal side effects.

## Graphical abstract

Iron oxide and silver sulfide nanoparticles are combined in optomagnetic nanocapsules, which can act as multimodal contrast agents for several imaging modalities (fluorescence imaging, magnetic resonance imaging, computed tomography, optical tomography, and photoacoustic imaging). More importantly, these nanocapsules enable real‐time subcutaneous thermal monitoring of magnetic hyperthermia processes, paving the way to fully controlled magnetic therapies.

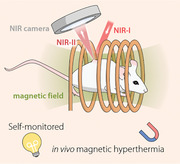

## 1 Introduction

Temperature and heat exchange are at the base of biological processes throughout the realm of nature. In reptiles, a difference of less than one degree in the egg incubation temperature determines the sex of the newborn.^[^ ^1^ ^]^ The swimming speed of ciliated and flagellated unicellular species is also temperature‐dependent.^[^ ^2^ ^]^ The human body is no exception. For instance, a temperature increase above the basal level could trigger physiological events such as modification of blood perfusion, alteration of cell dynamics, tumor matrix dismantling, blocking of DNA reparation mechanisms, increment of perfusion rates across cell membrane, and protein denaturation.^[^ ^3^, ^4^, ^5^, ^6^, ^7^ ^]^ As a matter of fact, our body can deliberately elevate its temperature to support immune response against a plethora of conditions, which we experience as fever.^[^ ^8^ ^]^ By the same token, activation of some of the abovementioned processes via localized heat delivery is the cornerstone of thermal therapies.^[^ ^9^, ^10^, ^11^ ^]^

Among these therapies, magnetic hyperthermia (MHT) achieves local temperature increase leveraging the ability of certain magnetic nanoparticles (MNPs) to generate heat when subjected to an alternating magnetic field.^[^ ^12^, ^13^ ^]^ The deep penetration of magnetic fields within the human body, together with the possibility of targeting MNPs to specific tissues, have enabled minimally invasive, local, and remotely activated heating showing high efficacy at the in vitro and in vivo levels.^[^ ^14^, ^15^, ^16^, ^17^, ^18^, ^19^, ^20^, ^21^, ^22^, ^23^ ^]^ Clinical trials have shown the efficacy of MHT to treat solid tumors, as well as to increase the usefulness of chemotherapy by enhancing drug permeability of cancer tissues.^[^ ^24^, ^25^ ^]^ Reaching high standards of efficacy and selectivity in MHT treatments requires full control over the remote heating process, which means precise localization of the MNPs and real‐time thermal feedback into the treated tissue.^[^ ^26^, ^27^ ^]^ Thus, efficient in vivo temperature reading methodologies allow assessing the efficacy and safety of the thermal treatment – a relevant limitation not yet addressed.

To tackle the first issue, in vivo localization of MNPs has been traditionally achieved via magnetic resonance imaging (MRI) and, more recently, magnetic particle imaging.^[^ ^28^, ^29^, ^30^, ^31^, ^32^ ^]^ The high resolution and penetration depths offered by these techniques are curbed by long acquisition times and the need for sophisticated equipment. Therefore, alternative imaging modalities have been proposed. To that end, the ability of MNPs to provide single and multimodal in vivo images has been exploited by combining other techniques like optical coherence tomography (OCT), MRI, computed tomography (CT), and photoacoustic (PA) imaging.^[^ ^33^, ^34^, ^35^, ^36^, ^37^, ^38^, ^39^ ^]^ MNPs have also been combined with near‐infrared (NIR)‐emitting nanoparticles (NPs) in a single hybrid structure, allowing multimodal imaging via NIR fluorescence and MRI.^[^ ^40^, ^41^ ^]^

On the other hand, precise, real‐time thermal feedback is needed for determining the delivered heat dose and for the dynamical setting of the treatment parameters.^[^ ^42^ ^]^ Usually, temperature monitoring during MHT is performed with infrared thermal cameras.^[^ ^43^ ^]^ Albeit being simple and cost‐effective, this approach provides information about the superficial temperature only (i.e., the skin), failing to give any reliable cue of the thermal state of deeper tissues. This is a pivotal aspect because, even during MHT of superficial tumors, the difference between intratumor and skin temperature could be greater than 10 °C.^[^ ^44^, ^45^ ^]^ Luminescence thermometry could afford a reliable solution to this conundrum.

Luminescence thermometry is a contactless thermometric approach that allows garnering thermal information about tissues in real‐time. This is accomplished by harnessing temperature‐dependent changes in the emission of luminescent species such as NPs, which effectively act as luminescent nanothermometers (LNThs).^[^ ^46^, ^47^, ^48^, ^49^ ^]^ LNThs have been employed to provide feedback during MHT both ex vivo and in vitro.^[^ ^50^, ^51^ ^]^ However, translation of these results to the in vivo level is no easy task. LNThs emitting in the second biological window (NIR‐II, 1000–1400 nm) are needed to ensure large optical penetration within tissues.^[^ ^52^, ^53^ ^]^ In addition, LNThs should have large thermal sensitivity (>1% °C^–1^), hence providing a thermal resolution (ideally, sub‐degree) smaller than the magnitude of the MHT‐induced tissue heating.^[^ ^53^ ^]^ In this vein, Ag_2_S NPs were recently shown to be prime candidates as in vivo LNThs, owing to their bright emission centered at approx. 1200 nm, excellent biocompatibility, good physicochemical stability, and, most importantly, temperature‐dependent optical properties.^[^ ^54^, ^55^, ^56^, ^57^, ^58^, ^59^, ^60^, ^61^, ^62^, ^63^ ^]^

**Figure 1:**
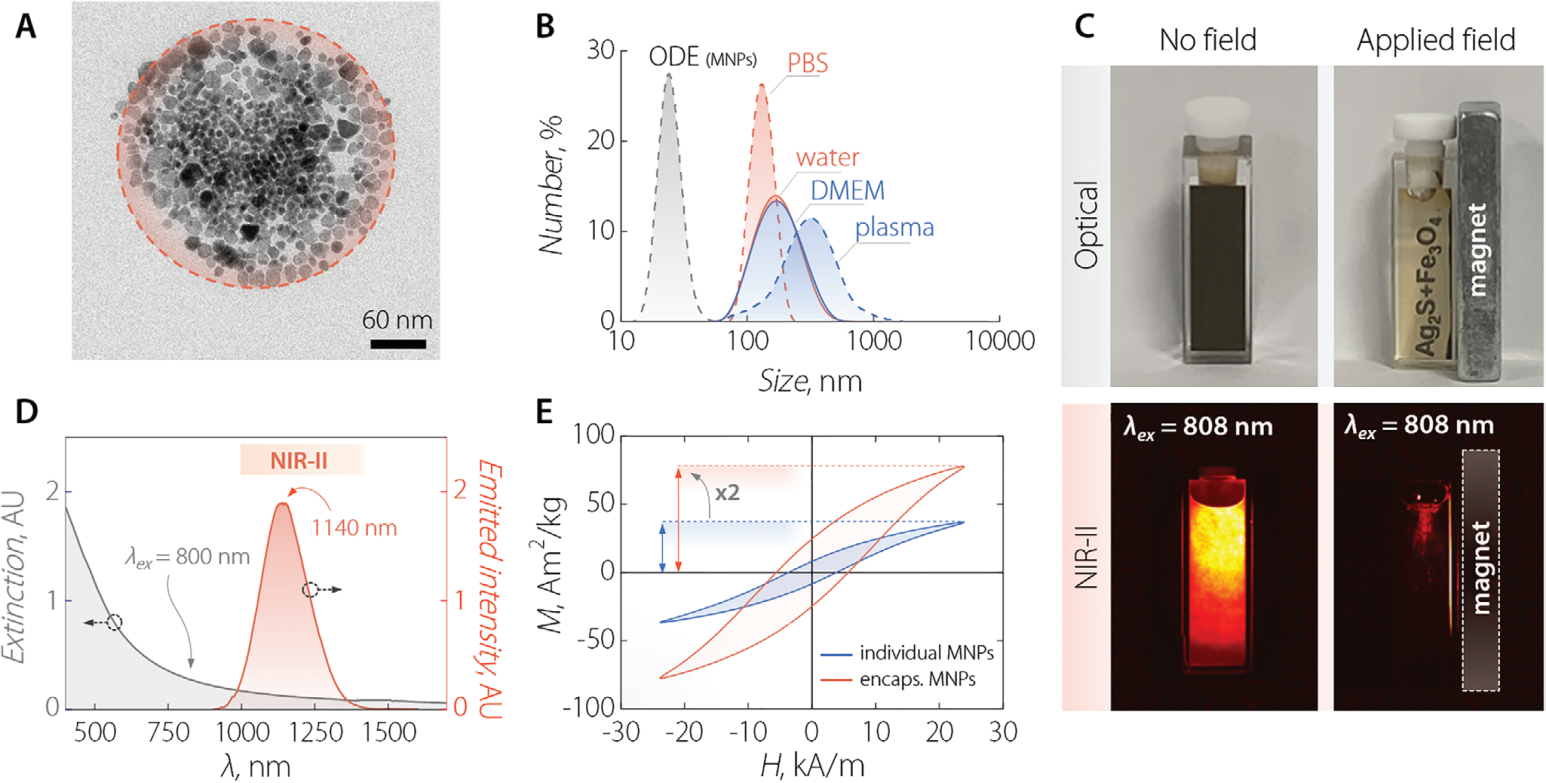
A) TEM image of a single NIR‐MNC consisting of Ag_2_S NPs and Fe_3_O_4_ MNPs co‐encapsulated by phospholipids. MNPs of larger size form a packed corona in the proximity of the nanocapsule's surface, surrounding smaller Ag_2_S NPs. B) Comparison between the hydrodynamic size distribution obtained via DLS on colloidal dispersions of MNPs in octadecene (ODE) and NIR‐MNCs in distilled water, phosphate‐buffered saline PBS 1×, DMEM, and blood plasma. C) Optical (top) and NIR‐II (bottom) fluorescence images of a colloidal dispersion of NIR‐MNCs in the absence (left) and presence (right) of a magnetic field gradient created by a neodymium magnet. NIR‐MNCs are dragged towards the magnet so the solution becomes clear and luminescence is only generated in the vicinity of the magnet. D) Room‐temperature extinction (gray line) and emission (orange line) spectra of a colloidal dispersion of NIR‐MNCs. The emission spectrum was obtained under 800 nm optical excitation. E) Comparison between the AC magnetic hysteresis loops obtained at 100 kHz and 24 kA m^−1^ from individual (blue line) and encapsulated (orange line) MNPs dispersed in 1‐octadecene and in double‐distilled water, respectively.

In this work, we propose a unique nanoplatform for in vivo heating, multimodal imaging, and real‐time thermal monitoring in the form of a NIR‐II‐emitting optomagnetic nanocapsule (NIR‐MNC) obtained by phospholipid encapsulation of Ag_2_S‐based NPs and MNPs (**Figure** **1**A).

## 2 Results and Discussion

The Ag_2_S‐based NPs consist of an Ag_2_S core passivated with layers of Ag_2_(S,Se),^[^ ^64^ ^]^ to improve the optical properties and resistance to oxidation (henceforth, we refer to them simply as Ag_2_S NPs). MNPs are superparamagnetic Fe_3_O_4_ NPs. The detailed experimental procedures for the NPs synthesis and encapsulation are reported in Section S1, Supporting Information. TEM observations revealed that, on average, within the nanocapsule multiple Ag_2_S NPs (mean diameter of 8.5 nm) are enclosed by Fe_3_O_4_ MNPs (mean diameter of 19 nm). This spatial arrangement of Fe_3_O_4_ and Ag_2_S NPs into the phospholipidic capsule can be explained tentatively in terms of size‐dependent interactions between the components of the asymmetric mixture, under the influence of the lipid membrane on the MNPs. This assumption was corroborated by Monte Carlo (MC) simulations in the canonical ensemble (Section S2, Figure S1, Supporting Information), wherein an experimentally based modeling of both the size‐dependent interparticle interactions and the modulation exerted by the lipid membrane on the MNPs was employed. The effective size of MNPs in organic media and NIR‐MNCs in different aqueous media of relevance for biomedical applications was studied by dynamic light scattering (DLS; Figure 1B; and Section S3, Supporting Information). On one hand, a size of ≈ 20 nm for MNPs dispersed in octadecene reflects their individual dispersions (the size obtained from TEM observations is 19 ± 3 nm) in organic media. On the other hand, typical values between 150 and 200 nm were obtained for nanocapsules dispersed in double distilled water, phosphate‐buffered saline (PBS 1×), and Dulbecco's modified Eagle medium (DMEM). In blood plasma, the size increased up to ≈ 300 nm, owing to the formation of a protein corona that, by modifying the surface charge, induces agglomeration of the nanocapsules.^[^ ^65^ ^]^

The low polydispersity index (PDI) values in aqueous media (Table S1, Supporting Information) indicated a relatively narrow size distribution, which, along with a lack of aggregation (save in the case of plasma), further corroborated the colloidal stability of the NIR‐MNCs. This stability resulted in comparable AC magnetization curves (Figure S2, Supporting Information) and specific absorption rate (SAR) values in different aqueous media (see Figures S3 and S4, Supporting Information). The efficient co‐encapsulation of MNPs and Ag_2_S NPs was confirmed by the application of a magnetic field gradient (Figure 1C). Upon placing a magnet on one side of a cuvette containing a dispersion of NIR‐MNCs, the colloid accumulated at the wall of the container under the drag of encapsulated Fe_3_O_4_ MNPs, leaving behind a clear solution. Fluorescence images (Figure 1C) showed no NIR emission from Ag_2_S NPs in the clear portion of the solution, evidencing the co‐encapsulation of the two moieties.

Notably, the absorption and emission spectra of NIR‐MNCs (Figure 1D) are, in terms of bandwidth and central wavelength, comparable to those previously reported for single Ag_2_S NPs^[^ ^66^ ^]^ and to those of Ag_2_S‐based NPs before encapsulation (Figure S5, Supporting Information), revealing that the NIR‐II optical properties are retained by the Ag_2_S NPs in the nanocapsule. Concerning the magnetic properties of NIR‐MNCs, room‐temperature magnetization cycles at 100 kHz and 24 kA m^−1^ obtained for encapsulated and as‐synthesized MNPs showed instead marked differences of the AC hysteresis loops (Figure 1E). Indeed, encapsulated MNPs featured larger coercive fields, magnetization values, and magnetic area than the ones dispersed in organic solvent. This magnetization difference (up to twofold larger; Figure S4, Supporting Information) can be understood in terms of ordered spatial distributions of MNPs^[^ ^67^ ^]^ that results in magnetising phenomena driven by nose‐to‐tail alignment of their magnetic moments. MC simulations detailed in Section S2 of the Supporting Information predict a spatial order of MNPs into NIR‐MNCs that, in combination with recent predictions,^[^ ^68^ ^]^ may explain the observed effective magnetization enhancement of encapsulated MNPs, and consequently their SAR values. Contrary to the case of a random MNP spatial distribution, the SAR values of NIR‐MNCs increased up to threefold with respect to the as synthesized MNPs.^[^ ^69^, ^70^ ^]^

**Figure 2:**
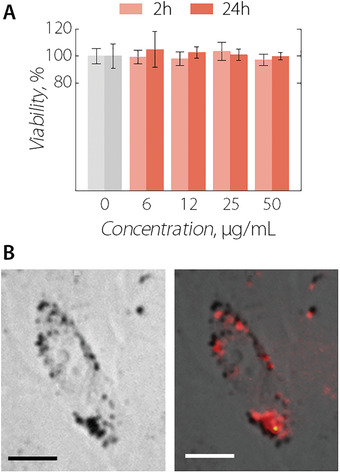
A) MCF‐7 cell viability after incubation with NIR‐MNCs at different extracellular concentrations. B) Optical (left) and NIR‐II fluorescence (right) images of a single MCF‐7 cancer cell after being incubated for 4 h with NIR‐MNCs at a concentration of 50 µg mL^−1^. The fluorescence image was obtained under broadband excitation (500–900 nm) and emission (1000–1500 nm). Scale bars in (B) are 5 µm.

Subsequently, the toxicity of the NIR‐MNCs at the in vitro level was evaluated upon incubating MCF‐7 cancer cells during 4 and 24 h with nanocapsule dispersions at different extracellular concentrations (0–50 µg mL^−1^; **Figure** **2**A). No cytotoxicity was observed at the tested conditions. Internalization of NIR‐MNCs by cells during incubation was corroborated by bright field microscopy (Section S6, Supporting Information) and NIR‐II fluorescence microscopy (Figure 2B). Cellular uptake quantification revealed an average mass per cell of 0.76 ± 0.01 pg_Fe_ and 1.04 ± 0.01 pg_Ag_ (Figure S7, Supporting Information) after 24 h of incubation. The marked cell internalization of NIR‐MNCs hinted at the amenability of these capsules for thermal treatments of tumors, because the heat generated would be delivered efficiently to the cancer cells.

**Figure 3:**
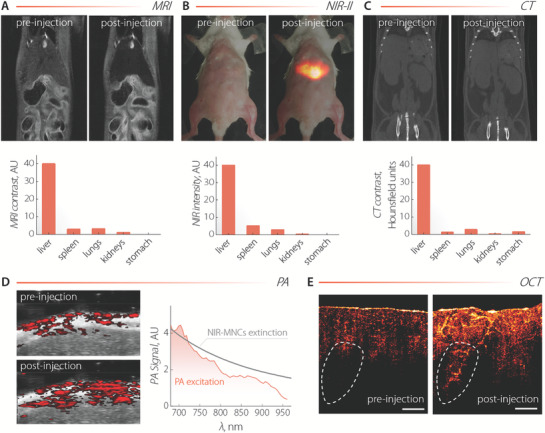
A) MRI, B) NIR‐II, and C) CT images of mice before and 30 min after administration of a dispersion of NIR‐MNCs (2 mg mL^−1^). 200 µL, 100 µL, and 300 µL of NIR‐MNCs dispersion were administered to each mouse, respectively. The biodistribution pattern obtained from each technique is also included. For MRI and CT, the patterns were obtained from in vivo images and the reported values are the product between the average contrast value for each organ obtained from image analysis and the weight of the organ. For NIR‐II imaging (λ_ex_ = 800 nm), the overall intensity from the different organs harvested after imaging (36 min in total; 30 min after injection + 6 min of imaging) was plotted. D) PA images of the liver (overlapped with ultrasound images) as obtained before and after intravenous (i.v.) injection of NIR‐MNCs (100 µL, 1.5 mg mL^−1^). λ_ex_ = 750 nm. The PA signal generated at the liver as a function of the excitation wavelength is also included (orange line), along with the absorption spectrum of NIR‐MNCs (gray line). E) OCT images of chicken breast tissue before and after injection of NIR‐MNCs (100 µL, 1.5 mg mL^−1^). The diffusion of NIR‐MNCs into the tissue makes possible the visualization of internal structures such as the vessel in the contoured areas. The scale bar is 1 mm.

After ascertaining the lack of appreciable toxicity, the capability of NIR‐MNCs to perform as multimodal imaging agents was assessed. In vitro tests were first conducted, which highlighted the possibility to use these capsules for MRI, NIR‐II fluorescence imaging, OCT, CT, as well as PA imaging (Section S7 and Figure S8, Supporting Information). With this knowledge at hand, we administered intravenously a NIR‐MNCs suspension at a concentration of 2 mg mL^−1^ to three different mice (**Figure** **3**A,B,C). Upon quantitative analysis of the images obtained via MRI, NIR‐II imaging, and CT before and after injection, a similar biodistribution pattern was retrieved, showing preferential accumulation of the nanocapsules at the liver along with non‐negligible collection at the lungs, kidneys, and stomach. For NIR‐II imaging, the full biodistribution pattern was obtained from the analysis of the ex vivo images of the organs. Prominent accumulation of NIR‐MNCs at the liver was also evidenced by an appreciable increase of the PA signal in correspondence with this organ (Figure 3D). To unequivocally correlate the enhancement of PA signal at the liver with the presence of NIR‐MNCs, we recorded an in vivo PA excitation spectrum. The resemblance of this spectrum with the extinction spectrum of NIR‐MNCs confirmed that the signal originated from the nanocapsules. 2D OCT images of ex vivo chicken breast tissue before and after injection of NIR‐MNCs were also acquired, and they showed a marked increase of OCT contrast due to the presence of the nanocapsules (Figure 3E). Interestingly, the diffusion of NIR‐MNCs within the tissue allowed to visualize deep vessels otherwise not observable (contoured area).

Altogether, the images included in Figure 3 demonstrate the potential of the NIR‐MNCs to act as multimodal contrast agents. This versatility allows selecting the imaging modality better suited for the specific application/tissue targeted as well as cross‐checking the interpretation of images obtained with different techniques.

**Figure 4:**
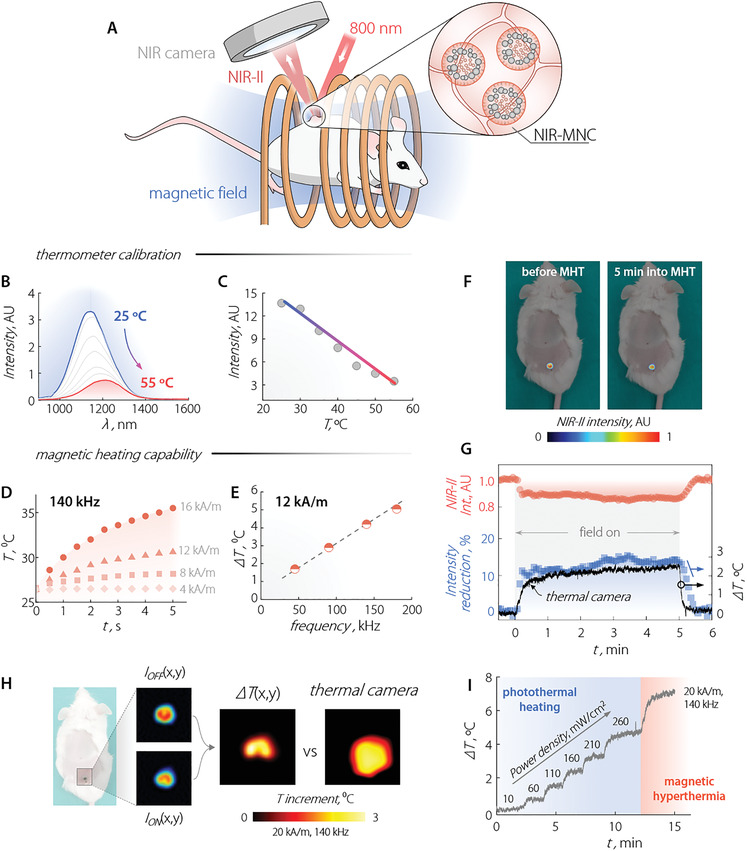
A) Scheme of the setup used for in vivo MHT experiments. B) NIR‐II emission spectra of a colloidal dispersion of NIR‐MNCs (in water) under a 1 mm layer of murine skin at different temperatures. λ_ex_ = 800 nm. C) Temperature dependence of the integrated emission intensity of the NIR‐MNCs under ex vivo murine skin. The temperature was recorded with the aid of a thermocouple. Symbols are data obtained from the analysis of the emission spectra and the solid line is a guide for the eye. D) Temporal evolution of the temperature of a colloidal dispersion of NIR‐MNCs in water for different AC magnetic field intensities with a frequency of 140 kHz. E) Temperature increment induced in a colloidal dispersion of NIR‐MNCs subjected to a 12 kA m^−1^ AC magnetic field for different frequencies. Solution temperature was registered by an optical fibre thermal sensor. F) NIR‐II fluorescence images of a CD1 mouse subjected to intradermal injection of NIR‐MNCs before and 5 min after the application of a 20 kA m^−1^ magnetic field of 100 kHz frequency. G) Temporal evolution of the intradermal NIR‐II emission intensity (orange) and the relative reduction in the NIR‐II intensity (blue) during the application of a 20 kA m^−1^, 100 kHz AC magnetic field for 5 min. The relative intensity reduction was converted to a relative change of the intradermal temperature using the calibration curve reported in (C). For the sake of comparison, in (F), the thermal readout obtained with an infrared thermal camera is also reported (black line). H) Intradermal thermal image obtained in the presence of a 20 kA m^−1^, 140 kHz AC magnetic as obtained from the NIR‐II fluorescence images obtained in the presence and absence of the magnetic field. Cutaneous thermal images acquired with an infrared thermal camera are also included. I) Temporal evolution of the intradermal temperature during the application of subsequent photothermal and hyperthermia processes as obtained from a thermal camera.

Last, the efficacy of NIR‐MNCs as thermally self‐monitored hyperthermia agents was assessed in a custom‐designed commercial equipment constituted of two coils connected to an AC generator that creates a homogeneous magnetic field at the mouse's position (**Figure** **4**A and Section S8, Supporting Information). To obtain a thermal read‐out, the temperature‐dependent variations of the NIR‐II emission band of Ag_2_S were harnessed (Figure 4B; and Sections S9 and Figure S10, Supporting Information). As we previously reported, the integrated intensity of emission, *I*, was expected to decrease linearly with the temperature.^[^ ^54^ ^]^ To thoroughly characterize the potential application of the Ag_2_S NPs in in vivo studies, we observed the thermal dependence of their luminescence spectrum under the presence of a biological tissue. More specifically, a 1‐mm layer of murine skin was placed atop a microchannel containing a dispersion of the NIR‐MNCs in water. The results are included in Figure 4C and they show that the luminescence could be effortlessly detected even at the highest tested temperatures. This through‐tissue calibration of the luminescent thermometer was preferred over a calibration in the absence of tissue since it better replicates the conditions of in vivo subcutaneous MHT monitoring (see Figure S11, Supporting Information and relative discussion). The relative thermal sensitivity (defined as $S_{r}=\left(\frac{1}{I}\right)\;\;\left|\frac{dI}{dT}\right|$) provided by the NIR‐MNCs was approximately 5.5% °C^–1^ in the 34–38 °C temperature range. This value is significantly larger than the thermal sensitivities achieved with other luminescent nanothermometers in this range.^[^ ^71^ ^]^ To the best of our knowledge, there is only one published study reporting on the use of luminescent thermometers (Nd^3+^‐doped fluoride NPs) supporting multimodal imaging.^[^ ^72^ ^]^ However, in that study by Wang et al., the relative thermal sensitivity was close to 2% °C^–1^ at room temperature and only MR and CT contrast capabilities were reported. The amalgamation of different moieties (MNPs and Ag_2_S NPs) with magnetic and optical properties in a single entity clearly allows achieving properties otherwise hardly attainable using these materials individually.

In addition to their thermal sensing ability, NIR‐MNCs generate heat when subjected to alternating magnetic fields of different intensities and frequencies (Figure 4D,E). The heating efficiency increases with the amplitude of the magnetic field, and a 9 °C temperature increase was achieved after 5 min for the highest tested magnetic field intensity (16 kA m^−1^, 140 kHz). As expected, a linear dependence of the magnetic‐field‐induced temperature increment on the frequency of the magnetic field (12 kA m^−1^) was also observed (Figure 4E).

Encouraged by these observations, we tested the prowess of NIR‐MNCs for thermally monitored in vivo MHT. To do so, we performed an intradermal injection of NIR‐MNCs (100 µL, 1.5 mg mL^−1^) in a CD1 mouse (Section S10, Supporting Information). The anesthetized animal was placed inside our custom‐built system (Figure 4A). The system has an optical window to acquire the fluorescence image of the mouse during the application of the magnetic field. The excitation intensity (λ_ex_ = 800 nm) was set to 10 mW cm^−2^ to keep laser‐induced heating at a minimum. NIR‐II fluorescence images of the mouse before and 5 min into the application of an AC magnetic field (20 kA m^−1^, 140 kHz) showed a similar distribution of NIR‐MNCs (Figure 4F). The partially quenched emission observed 5 min into the MHT treatment indicated local heating at the site of the intradermal injection. The temporal evolution of the NIR‐II signal from NIR‐MNCs (Figure 4G) showed a relative decrease of ≈ 15% during the application of the magnetic field. Upon switching off the applied field, the NIR‐II signal recovered its original value. The drop in NIR‐II intensity was translated to temperature units using the calibration curve reported in Figure 4C, resulting in a measured increase of the temperature close to 2.5 °C. The uncertainty of the thermal readout provided by the NIR‐MNCs was determined to be 0.2 °C: This conservative estimate was obtained from the difference between the maximum and minimum temperature value during the first 30 s of irradiation in the absence of applied AC magnetic field. The evolution of the local temperature during the MHT treatment obtained via luminescence thermometry agreed well with the one measured with a thermal camera. Such agreement was expected because of the superficial nature of the intradermal injection, and it constitutes a solid proof of the reliability of the luminescence thermometric approach. Indeed, the reduced thickness of the dermis (<1 mm) allows efficient heat diffusion so that a relatively small average temperature difference of 0.4 °C exists between the injection site and the external surface, although with larger, yet expected, differences during the heating and cooling transients (see Figure S12, Supporting Information, and discussion therein).

However, in the case of deeper tissues/organs, only NIR‐MNCs provide a temperature readout close to the real value. Ex vivo results included in Section S11, Figure S13, Supporting Information corroborate the superiority of approaches based on NIR‐II emitting LNTh compared to the use of a thermal camera to gain knowledge about the thermal state below skin. From a technical viewpoint, real‐time readout of the magnetically activated hyperthermia has the benefit of providing a useful feedback to adjust the AC magnetic field so as to set the most efficient thermal exposure for tumor treatment, minimizing the heating time.

Importantly, our experimental setup allowed obtaining the first‐ever subcutaneous, in vivo 2D thermal images during MHT. The generic thermal image (Δ*T*(*x*,*y*)) was retrieved from the NIR‐II images acquired in the absence (*I*_off_(*x*,*y*)) and in the presence (*I*_on_(*x*,*y*)) of the magnetic field. By applying the calibration obtained under ex vivo conditions to each pixel, the difference between the initial and final temperature at each point was retrieved and a spatial profile of the temperature could be built (Figure 4H). Comparison of the 2D thermal maps obtained with this approach and with a thermal camera highlights that the latter method shows a larger spatial spreading of the temperature. This is the result of heat conduction from the particles within the tissues. NIR‐MNCs, instead, only provide thermal reading at the site where they are present.

Interestingly, thanks to the strong absorption from both Ag_2_S NPs and MNPs,^[^ ^73^, ^74^ ^]^ NIR‐MNCs can also efficiently convert light‐to‐heat, making them suitable candidates for combined photothermal heating and magnetic hyperthermia.^[^ ^75^, ^76^ ^]^ Under continuous irradiation at a power density of 10 mW cm^−2^ (the one used for fluorescence imaging), a temperature increase of (0.10 ± 0.06) °C was observed. However, increasing the power density of the excitation light above that threshold, the intradermal temperature was elevated by an amount dependent upon the power density itself (19 °C W cm^−^^2^)—Figure 4I). When the AC magnetic field was switched on, the magnetically induced heating added to the one generated via laser irradiation. In our experimental conditions, combined photothermal heating (laser power density of 260 mW cm^−2^) and magnetic hyperthermia (20 kA m^−1^, 140 kHz) led to an overall temperature increase close to 7 °C. The combination of photothermal heating and MHT opens new horizons for advanced thermal therapies where, for example, the sub‐tissue temperature can be orthogonally modulated by tuning of laser intensity and magnetic field amplitude/frequency.

## 3 Conclusions

We have synthesized optomagnetic nanocomposites by phospholipid‐mediated co‐encapsulation of NIR‐II‐emitting fluorescence nanothermometers (Ag_2_S nanoparticles) and heating mediators (Fe_3_O_4_ magnetic nanoparticles). The nanocapsules dispersed in water featured improved magnetic properties compared to as‐synthesized Fe_3_O_4_ magnetic nanoparticles dispersed in octadecene before encapsulation, likely due to collective magnetic effects. The capsules can act as sub‐tissue multimodal contrast agents for magnetic resonance imaging, computed tomography, infrared fluorescence imaging, photoacoustic imaging, and optical computed tomography. Most importantly, the developed optomagnetic capsules enable real‐time thermal feedback during in vivo magnetic hyperthermia with a thermal resolution of 0.2 °C, thanks to the thermal sensing ability of Ag_2_S nanoparticles. Furthermore, the use of these novel composites allows building 2D thermal maps of the treated location at the subcutaneous level: an actual “on‐site” thermal scan synergic to the hyperthermia treatment. The herein reported results constitute a crucial step toward fully controlled magnetic hyperthermia treatments, where collateral damages deriving from overheating or heating of healthy tissues will be avoided.

## Conflict of Interest

The authors declare no conflict of interest.

## Supporting information

Supporting Information

## Data Availability

The data that support the findings of this study are available from the corresponding author upon reasonable request.
