# Supplementary material for: Infrared‐Emitting Multimodal Nanostructures for Controlled In Vivo Magnetic Hyperthermia
Source: Adv Mater. 2021 Jun 12;33(30):2100077. doi: 10.1002/adma.202100077 (PMC11468761; doi:10.1002/adma.202100077)
Supplement: Supplementary file 1 — Supporting Information [file ADMA-33-2100077-s001.pdf]

© 2021 Wiley-VCH GmbH

ADVANCED MATERIALS

Supporting Information

for *Adv. Mater.,* DOI: 10.1002/adma.202100077

Infrared-Emitting Multimodal Nanostructures for Controlled In Vivo Magnetic Hyperthermia

Erving Ximendes, Riccardo Marin, Yingli Shen, Diego Ruiz, Diego Gómez-Cerezo, Paloma Rodríguez-Sevilla, Jose Lifante, Perla X. Viveros-Méndez, Francisco Gámez, David García-Soriano, Gorka Salas, Carmen Zalbidea, Ana Espinosa, Antonio Benayas, Nuria García-Carrillo, Lorena Cussó, Manuel Desco, Francisco J. Teran, Beatriz H. Juárez, and Daniel Jaque**

Copyright WILEY-VCH Verlag GmbH & Co. KGaA, 69469 Weinheim, Germany, 2018.

Supporting Information

Infrared-emitting multimodal nanostructures for controlled in vivo magnetic hyperthermia.

Erving Ximendes,† Riccardo Marin,† Yingli Shen, Diego Ruiz, Diego Gómez-Cerezo, Paloma Rodríguez-Sevilla, Jose Lifante, Perla X. Viveros-Méndez, Francisco Gámez, David García-Soriano, Gorka Salas, Carmen Zalbidea, Ana Espinosa, Antonio Benayas, Nuria García-Carrillo, Lorena Cussó, Manuel Desco, Francisco J. Teran, Beatriz H. Juárez and Daniel Jaque.**

Table of Contents

S1. Synthesis of nanoparticles (NPs) and encapsulation.	S-2
S2. Monte Carlo simulations details.	S-3
S3. DLS measurements.	S-7
S4. Magnetic measurements.	S-8
S5. Optical properties of Ag_2S nanoparticle before and after encapsulation.	S-10
S6. *In vitro* experiments: cell labelling and cytotoxicity assays.	S-11
S7. Use of NIR-MNCs as multimodal contrast agents.	S-12
S8. System for magnetic hyperthermia measurements.	S-14
S9. Calibration of the luminescence thermometer.	S-15
S9. *In vivo* magnetic hyperthermia experiments.	S-17
S10. *Ex vivo* experiments with and without tissue.	S-18
S11. References.	S-19

S1. Synthesis of nanoparticles (NPs) and encapsulation.

Chemicals. Silver (I) diethyldithiocarbamate (AgDDTC, 99%), 1-dodecanethiol (DDT, >98%), toluene (TOL, 99.8%), acetone (technical grade), oleylamine (OLA, 70%), sulfur powder (S, synthesis grade), ethanol (EtOH, 96%), chloroform (99.8%), hexane (reagent grade), selenium powder (Se, 99.99%), trioctylphosphine (TOP, 97%), Iron(III) acetylacetonate ([Fe(acac)$_3$], 97%), oleic acid (OA, 90%), 1,2-dodecanediol (DDD, 97%), 1-octadecene (ODE, 90%), dimethylsulfoxide (DMSO, >99%) and 1,2-dipalmitoyl-sn-glycero-3-phosphoethanolamine-N-[methoxy(polyethylene glycol)-2000] ammonium salt (PE:PEG2000). All chemicals were used without further purification. All chemicals were purchased from Sigma-Aldrich except PE:PEG2000 that was purchased from Avanti Polar Lipids.

Synthesis of the Ag_2S-based NPs. These NPs consist of a Ag_2S core coated with a Ag_2(S,Se) shell, as described elsewhere.[1] For the synthesis, 0.2 mmol of AgDDTC, 60 mmol of DDT and 10 mL of TOL were mixed in a three-neck round-bottom flask. The mixture was heated to 100 °C. Upon complete dissolution of the silver precursor, the solution turned to a bright yellow color and 100 µL of a solution of 0.4 mmol of S powder in 1 mL of OLA was swiftly injected. After 5 min, 100 µL of a 1 M solution of Se powder in TOP was injected into the NPs dispersion. The solution was left to react for 10 min and then let to cool naturally to room temperature. Several washing steps were carried out with acetone and chloroform. After the washing steps, the NPs were dispersed in chloroform.

Synthesis of Fe_3O_4 magnetic nanoparticles (MNPs). MNPs were synthesized using the method reported by Sun et al. with some modifications.[2] Briefly, [Fe(acac)$_3$] (3.53 g, 10 mmol), OA (8.47 g, 30 mmol), and DDD (4.05 g, 20 mmol) were dissolved in ODE (100 mL), in a three-neck round-bottom flask with mechanical stirring under a flow of N_2 at atmospheric pressure. The mixture was heated at a constant rate of 2 °C/min until the temperature reached 190 °C. The reaction mixture was kept at that temperature for 1 h, subsequently heated until reflux at a constant rate of 3° C/min, and kept at that temperature for 30 min. Then, the reaction was stopped allowing the mixture to cool down to room temperature. Finally, the solution was extracted in hexane, washed by centrifugation with EtOH repeatedly and stored in hexane.

Encapsulation in phospholipids: preparation of NIR-MNCs. Out of the different strategies to encapsulate NPs,[3,4] the solvent exchange method was employed.[5,6] The NPs must be washed twice to remove any excess of ligands prior to their encapsulation. Washing steps consist on centrifugation in the presence of anti-solvents (acetone, ethanol) and re-dispersion in chloroform. In a typical experiment, 8 mL of a 1.8-mg/mL dispersion of Ag_2S NPs and 4 mL of a 2-mg/mL Fe_3O_4 MNPs dispersion were mixed in chloroform and the mixed dispersion was concentrated to a volume of 4 mL by centrifugation. After that, 2 mL of a 15-mg/mL solution of the PE:PEG2000 were added. The mixture was left for 30 min in a mechanical agitator to promote the interaction between the NPs and the phospholipids. After that, 40 mL of DMSO were

added to the chloroform mixture and after agitation and sonication, the chloroform was allowed to evaporate at room temperature overnight. Any remaining chloroform was evaporated using a gentle N_2 flow. Once the chloroform was completely removed, aliquots (20 mL) were taken from the DMSO batch. Each aliquot was diluted to half the concentration with DMSO. From the diluted portion, 5 mL aliquots were transferred to glass vials. Then, 15 mL of milliQ water were quickly added to each aliquot and the mixture was sonicated for 15 s. The formation of encapsulates is accompanied by a slight increase in temperature, that can be felt on the surface of the glass vial. Upon water addition, the mixture should appear highly transparent. A strongly light scattering mixture after water addition signifies big vesicles with low encapsulation efficiency. After water addition to all diluted aliquots, the DMSO is removed from the sample by centrifugation at 9000 rpm for 30 min and the pellets are redispersed in milliQ water. Several washing procedures consisting of re-dispersion of the sediments in water, sonication and further centrifugation were performed.

S2. Monte Carlo simulations details.

Monte Carlo simulations were carried out in the canonical (*NVT*) ensemble under spherical confinement with a home-built code. The composition of the idealized colloidal mixture and the size of both the NPs and the NIR-MNCs were taken from TEM and DLS experiments, respectively. The initial configurations consisted of a randomly distributed equimolar mixture, with a total number of NPs fixed at $N = 300$ at a constant temperature of $T = 300$ K. The capsule diameter was fixed to 250 nm and those of the colloidal moieties were $\sigma_1 = 8.5$ nm for Ag_2S NPs and $\sigma_2 = 19$ nm for Fe_3O_4 MNPs. Approximately 10^{10} cycles were used for equilibration and 10^7 to evaluate the averages in the production run. Each cycle consisted of one attempt of translation of each particle in the mixture.

The interaction between a pair of colloids whose centers are at a distance *r* apart (U) was comprised by an attractive van der Waals interaction, $\mathrm{U_{vdW}}$, plus a repulsive Yukawa interaction, $\mathrm{U_Y}$, of the general form:[7]

$$\mathrm{U_{vdW}}(r) = -\frac{A}{12}\left[\frac{\sigma^2}{r^2} + \frac{\sigma^2}{r^2-\sigma^2} + 2\ln\left(\frac{r^2-\sigma^2}{r^2}\right)\right] \quad \text{(S1)}$$

and

$$\mathrm{U_Y}(r) = 16\pi\epsilon\epsilon_0\sigma^2\left[\frac{\mathrm{k_B T}}{\mathrm{e_0}}\tanh\left(\frac{\mathrm{e_0}\zeta}{4\mathrm{k_B T}}\right)\right]^2\frac{\exp[-\kappa(r-\sigma)]}{r} \quad \text{(S2)}$$

where ϵ and ϵ_0 are the dielectric constant of the dispersing medium and the dielectric constant of the vacuum, respectively, *A* is the Hamaker constant, $\mathrm{e_0}$ is the elementary charge, ζ is the ζ-potential, κ is the inverse Debye length, $\mathrm{k_B}$ is the Boltzmann constant, and T them temperature of the system. For crossed interactions, $A_{12} = (A_1 \cdot A_2)^{1/2}$ and $\sigma_{12} = (\sigma_1 + \sigma_2)/2$. Hamaker constants were taken from the literature for both Ag_2S and Fe_3O_4 MNPs.[8, 9] The ζ-potential value for Ag_2S NPs in water was measured after a ligand-exchange procedure with polyethylene glycol methylether thiol (PEG-SH), as described in [1]. While the ζ-potential for the Ag_2S NPs was experimentally measured (33.5 mV), the value for the MNPs was incorporated into the model by assuming a typical value of $\kappa\sigma_1 = 0.15$, and interpolating the experimental data for hydrophobic alkane chains at pH = 7 from [10]. Irreversible coagulation was prevented by adding a hard potential at a surface-to-surface distance, typical of coated nanoparticles brushes, of 4 nm.

Furthermore, for the membrane-particle interaction, the sphere-plate geometry was selected. The electrostatic part was considered in the extended linear superposition approximation (LSA):[11]

$$\mathrm{U_{vdW}}(H) = -\frac{A}{6}\left[\frac{\sigma}{2\mathrm{H}} + \frac{\sigma}{2(\mathrm{H}+\sigma)} + 2\ln\left(\frac{\mathrm{H}_{ss}}{\mathrm{H}+\sigma}\right)\right] \quad \text{(S3)}$$

and

$$U_Y(H_{ss}) = U_{LSA}(H_{ss}) + U_{LSA}(H_{ss}+\sigma) + \frac{64\pi\epsilon\epsilon_0}{\kappa}\left[\frac{k_BT}{e_0}\tanh\left(\frac{e_0\zeta_M}{4k_BT}\right)\right]\left[\frac{k_BT}{e_0}\tanh\left(\frac{e_0\zeta}{4k_BT}\right)\right](-\exp[-\kappa H_{ss}] + \exp[-\kappa(H_{ss}+\sigma)]) \quad \text{(S4)}$$

with

$$U_{LSA}(x) = 32\pi\epsilon\epsilon_0\sigma\left[\frac{k_BT}{e_0}\tanh\left(\frac{e_0\zeta_M}{4k_BT}\right)\right]\left[\frac{k_BT}{e_0}\tanh\left(\frac{e_0\zeta}{4k_BT}\right)\right]\exp[-\kappa x] \quad \text{(S5)}$$

ζ_M is the ζ-potential of the membrane, and H_{ss} is the surface-surface distance. Because of the similar chemical composition of the magnetic NPs coating and the phospholipids hydrophobic chains, an additional attractive hydrophobic interaction was included. We followed an expression of the form:

$$U_{HB}(H_{ss}) = -\frac{K}{6}\left[\frac{\sigma}{2H} + \frac{\sigma}{2(H+\sigma)} + 2\ln\left(\frac{H_{ss}}{H+\sigma}\right)\right] \quad \text{(S6)}$$

where the hydrophobic constant was chosen to be $K = 100\cdot10^{-21}$ J.[12]

Additionally, the influence of the magnetic field on the NPs distribution within the confining volume was studied upon the incorporation of a magnetic dipole interaction. For a given superparamagnetic system which possesses a field-induced saturation magnetic dipole m_S, this interaction takes the general form [13]:

$$U_{MD}(r) = \frac{m_S^2\mu_0}{4\pi r^3}(1 - 3\cos^2\theta), \quad \text{(S7)}$$

where μ_0 is the magnetic permeability of the vacuum, and θ is the angle between the magnetic field and center-to-center distance between arbitrary particles. The saturation value of the magnetic dipole for Fe_3O_4 NPs was fixed at a reasonable value of $m_S = 4.5\cdot10^5\cdot V_P$ $A\cdot m^2$,[14] with V_P being the particle volume.

The main results of the simulations are summarized in **Figure S1**. In **Figure S1A**, the energy profile of the colloid-colloid interaction is plotted together with the hard-core potential included in the overall potential, whose position is denoted by vertical lines. In **Figure S1B**, a quantitative description of the particle distribution with and without magnetic field (N = 300) was extracted with the calculation of the radial density distribution function ρ. This function provides the number of particles of a certain type within a spherical shell of volume $(4/3)\pi[(\delta+d\delta)^3-\delta^3]$, with δ being the radial distance from the origin. In **Figure S1C**, the equilibrium configurations for the simulations in the absence and presence of a magnetic field are presented as snapshots. From these pictures, it is apparent that in field-free conditions the MNPs are mainly distributed in the peripheral part of the capsule, while Ag_2S NPs are distributed throughout the sphere, in agreement with the experimental results. In the presence of a magnetic field, although the function ρ shows a similar behaviour, it is evident that MNP magnetic moments are aligned along the applied magnetic field direction in a chain-like magnetic ordering. Since the MNPs distribution is mainly a consequence of the interactions with

the phospholipids and the magnetic field, the modulation provoked by the Ag_2S-MNPs interaction is not expected to result in a substantial modification of the circular structure of the MNPs found in the simulation. This feature may be intimately related with the magnetization enhancement experimentally observed and detailed in the main text.

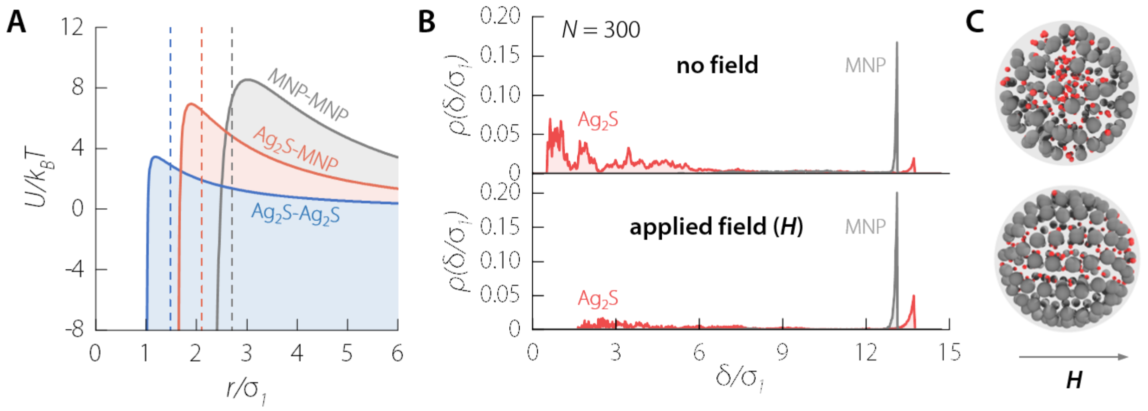

Figure S1. A) Energy profile of the colloid-colloid interaction. Vertical lines denote the position of the hard-core potential. **B)** Radial density distribution functions ρ as a function of the radial distance to the origin δ for number of particles, N = 300 and **C)** corresponding snapshots of the NPs equilibrium distribution in the absence (top) and in the presence (bottom) of applied magnetic field.

S3. DLS measurements.

To determine the effective size of the magnetic colloids in different solvents, dynamic light scattering (DLS) measurements were performed by using a Zetasizer Nano ZS90 (Malvern Instruments) equipped with a 4.0-mW He−Ne laser operating at 633 nm as energy source, with an angle of 173˚ between the incident beam and the avalanche photodiode detector. The measurements were performed with an automatic scan time, and three scans per measurement. Different aqueous media were employed for measuring the colloidal properties of the capsules: doubly distilled water (DDW), phosphate buffer saline (PBS 1x), cell culture medium (75% commercial DMEM (Biowest), 15% PBS and 10% FBS), and blood plasma (75% dilution of commercial blood plasma, 25% PBS 1x) (Biowest, Ref.: FTS4180an). In order to disperse the NIR-MNCs at 1 g_{Fe}/L in different media, we centrifuged 40 µL of nanocapsules dispersed in DDW using a Galaxy Mini Centrifuge (VWR) at 600 rpm during 15 min. Then, 30 µL of supernatant was removed, and 30 µL of the biological medium of choice was added to redisperse the NIR-MNCs by using a vortex.

Table S1: ζ-potential and number-weighted hydrodynamic sizes with the corresponding standard deviations, along with polydispersity indexes (PDI), obtained from DLS measurements of the studied MNPs and NIR-MNCs dispersed in different media.

**Sample**	***Medium***	***Hydrodynamic size*, nm**	***PDI***	***ζ-potential*, mV**	***pH***
Individual MNPs	1-octadecene	25 ± 4	0.58	-	-
NIR-MNCs	DDW	162 ± 18	0.15	-27 ± 8	6.7
NIR-MNCs	PBS 1x	158 ± 17	0.15	-12 ± 7	7.4
NIR-MNCs	DMEM	162 ± 21	0.22	-8 ± 1	8.8
NIR-MNCs	Blood plasma	286 ± 66	0.84	-6 ± 1	8.4

S4. Magnetic measurements.

DC/AC Magnetization Measurements. Magnetometry measurements were carried out at room temperature and at different iron concentration in the frequency range 30-300 kHz, and a magnetic field amplitude of 24 kA/m. AC hysteresis loops were traced with a commercial inductive magnetometer (AC Hyster, Nanotech Solutions). The values of AC magnetization were normalized to the mass of magnetic element (i.e., iron). AC hysteresis loop measurements consists in three repetitions to obtain average and standard deviation of the magnetic area values. Afterwards, SAR values were calculated according to $SAR = A \cdot f$, where A is the magnetic area and f is the AC magnetic field frequency.

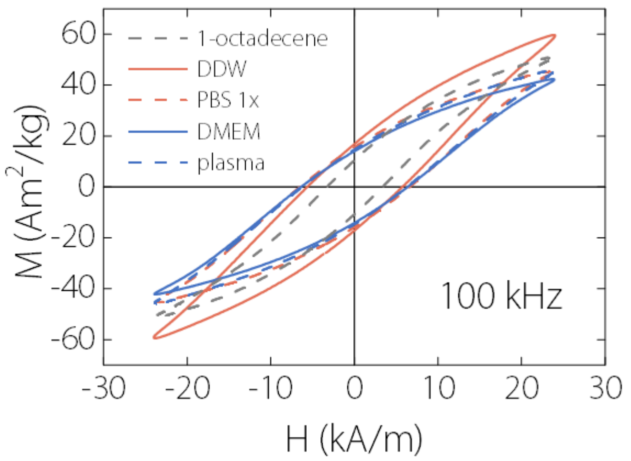

Figure S2. Comparison of mass-normalized AC hysteresis loops obtained from individual MNPs dispersed in 1-octadecene (grey dashed line), and encapsulated MNPs dispersed in double-distilled water (orange solid line), PBS 1x (orange dashed line), DMEM (blue solid line), and blood plasma (blue dashed line). The iron content in all colloidal dispersions was 1 g_{Fe}/L and field conditions of 100 kHz and 24 kA/m.

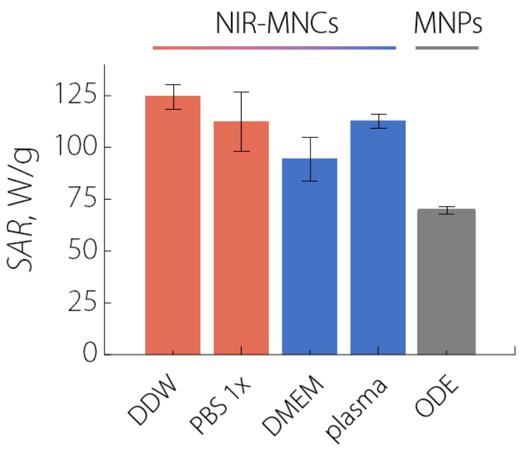

Figure S3. Comparison of SAR values obtained from measurements shown in **Figure S2**: individual MNPs dispersed in 1-octadecene, and encapsulated MNPs dispersed in double-distilled water, PBS 1x, cell culture medium, and blood plasma. The iron content in all colloidal dispersions was 1 g/L and field conditions of 100 kHz and 24 kA/m.

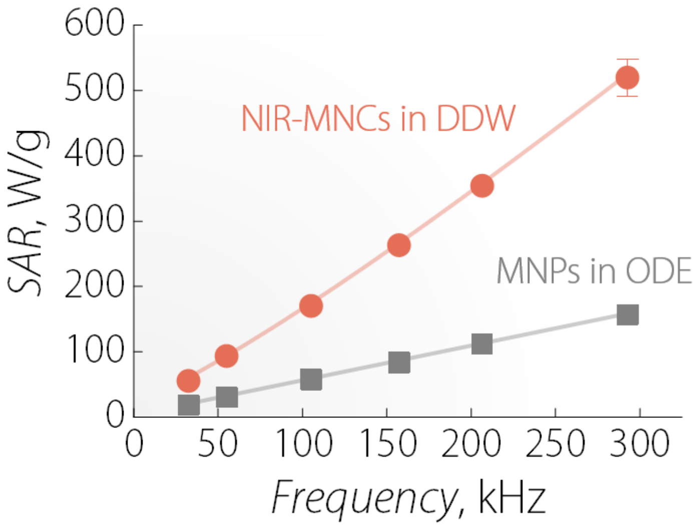

Figure S4. Comparison of SAR values obtained from MNPs into NIR-MNCs dispersed in double-distilled water (orange) and individual MNPs dispersed in 1-octadecene (grey). The iron content in the colloidal dispersions was 2 g_{Fe}/L and field conditions of 100 kHz and 24 kA/m. Lines are guides for the eye.

S5. Optical properties of Ag_2S nanoparticle before and after encapsulation.

In **Figure S5**, the absorption and emission spectra of Ag_2S NPs before and after encapsulation in phospholipids are reported. The spectra were normalised to better appreciate changes in the spectral shape. The emission intensity of Ag_2S NPs after encapsulation decreases by 50-70% of the initial signal. This reduction in intensity can be due to both a low encapsulation efficiency, as previously reported by other authors,[15] and a decrease in the quantum yield of the encapsulated Ag_2S NPs. The latter effect could result from a modified surface chemistry of the NPs upon phospholipid-mediated transfer to water. Upon comparing the shape of the emission spectra before and after encapsulation, it was observed that the bandwidth and central wavelength of the emission band were not substantially affected by the encapsulation process. We should finally note that the emission peak of the Ag_2S NPs herein presented is centered at 1200 nm, whereas the one of the Ag_2S NPs discussed in the main text have an emission band peaking at around 1150 nm. This batch-to-batch variability of the peak position was expected, due to slight differenced in the Ag_2S core size and Ag_2Se shell thicknes.

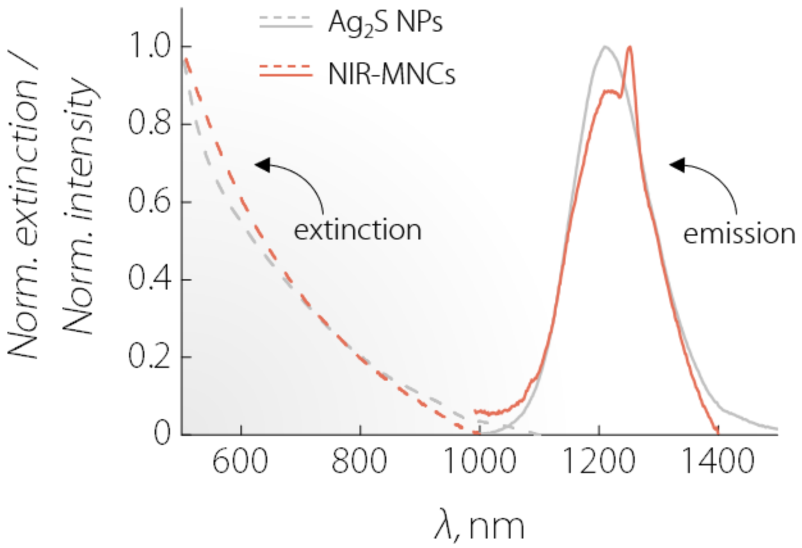

Figure S5. Absorption (dahsed lines) and emission (solid lines) spectra of Ag_2S NPs before (grey lines) and after encapsulation (orange lines). The spectra were measured under 800-nm excitation in tetrachloroethylene (TCE) and in water before and after encapsulation, respectively. The modulation of the emission peak of NIR-MNCs follows from reabsorption of the luminescence signal from water and the iron oxide nanoparticles in the capsule.

S6. *In vitro* experiments: cell labelling and cytotoxicity assays

Cell labelling and viability tests. MCF-7 cells (human breast cancer cell line) were cultured in Dulbecco's modified Eagle's medium (DMEM) supplemented with 10% FBS, 1% penicillin and 1% glutamine in an incubator (37 ºC, 5% CO_2). Cells were seeded into 24-well plates and incubated with NIR-MNCs at different MNCs extracellular concentrations (0 to 50 µg/mL) for 4 and 24 h in DMEM medium. At the end of the incubation, the medium was removed and the cells were washed with culture medium. Treated cells were incubated with Resazurin dye-based solution and DMEM without red phenol for 3-4 h. The reagent was then transferred to a 96-well plate and analyzed with a fluorescence microplate reader (Ex/Em = 540/590 nm, Biotek Instruments). Cytotoxicity was determined by comparison with control cells. All reported experiments (**Figure 2A**) were performed in triplicate.

Bright field microscopy. Cells were observed via bright field microscopy on a Leica 2500 fluorescence microscope. Results are included in **Figure S6**.

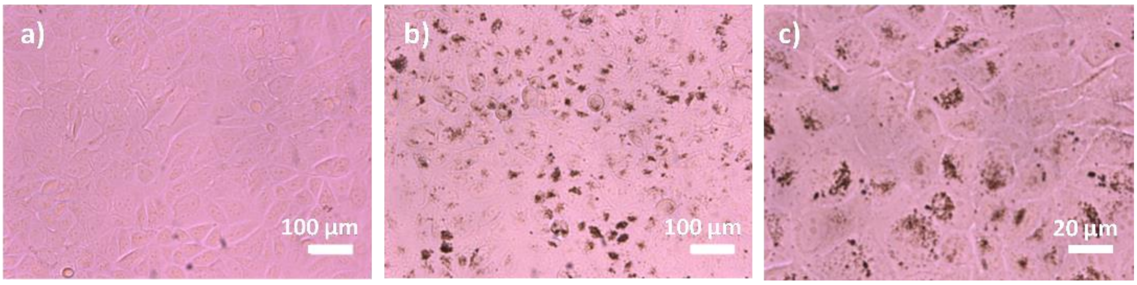

Figure S6. Cells incubated with NIR-MNCs visualized by bright-field microscopy. **a)** Control cells, **b, c)** cells incubated with NIR-MNCs for 4 h (at extracellular concentration 50 µg/mL).

Cellular uptake quantification. Cellular uptake of NIR-MNSs were evaluated by inductively coupled plasma (ICP-OES). After incubation, cells were collected and diluted with a solution of aqua regia. A series of iron and silver standard solutions were initially measured for calibration. Results are shown in **Figure S7.**

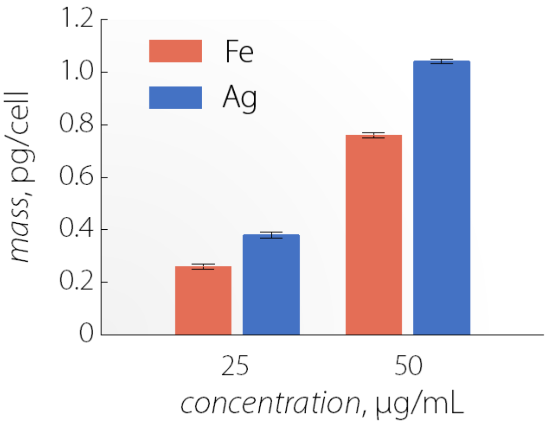

Figure S7. Cellular uptake of NIR-MNCs. Cellular uptake of the nanocapsules as a function of extracellular concentration (mass of Fe and Ag per cell) after 24 h of incubation.

S7. Use of NIR-MNCs as multimodal contrast agents.

Magentic resonance imaging and computed tomography protocol. Magnetic resonance images were acquired in a 7 Tesla BioSpec 70/20 (Bruker, Germany) using a transmit-receive volume coil. To calculate the relaxivity (*r2*) of the nanoparticles, T2 maps of pure water and dilutions of the sample in water were acquired at different echo times. A multi gradient echo (MGE) sequence was used with the following parameters: TR = 200 ms; TE = 25 echo times from 6.4 ms to 159.6 ms with an echo spacing of 6.4 ms; slice thickness = 1 mm; 1 average; FOV = 67.4×57.6 mm; matrix = 128×128. r2 was obtained from the slope of the line that fits the relationship between the concentration and the inverse of T2. For the calculation of T2 maps, the pixel intensity of each image was fitted to $S = S_0 \exp{(-TE/T2)}$ where *S* is the intensity of the image at each TE. *In vivo* T2 images were acquired in a 30-g female C57BL/6 mouse before and after the intravenous administration of 0.2 mL of a 2-mg/mL suspension of NIR-MNCs. A Turbo RARE (Rapid Acquisition with Relaxation Enhancement) sequence was used with the following parameter: RARE factor = 8; echo spacing = 8 ms; TR/TE = 730/24 ms; 8 averages; matrix = 256×256; FOV = 40x40 mm; slice thickness = 1 mm; 9 slices. Computed tomography (CT) images were acquired with a SuperArgus PET-CT (Sedecal, Spain). Both NIR-MNCs dilutions and *in vivo* images were acquired at 340 µA and 40 kVp. Images were reconstructed with a Feldkamp, Davis, and Kres (FDK) algorithm. *In vivo* CT images were acquired in a 30-g female C57BL/6 mouse before and after of intravenous administration of 0.3 mL of 2 mg/mL suspension of NIR-MNCs.

Photoacoustic imaging. In vivo photoacoustic (PA) imaging was performed by suing the VevoLAZR-X system (Fujifilm VisualSonics Inc.). This photoacoustic imaging commercial system uses, as a light source, an optical parametric oscillator (OPO) that is optically pumped by the 532 nm laser radiation generated by a frequency-doubled Nd:YAG laser. The repetition frequency of the train of 5 ns pulses was set to 10 Hz. The hand-held linear array probe (15 MHz center frequency; 256 acoustic elements) was mechanically coupled to two fiber arrays at both sides of the acoustic aperture of the transducer. These arrays were coupled to the OPO. The illumination angle (tilt angle) of the optical fibers was 30º. Ultrasound and PA images were recorded at a frame rate of 5 Hz.

Optical coherence tomography. One hundred µL of a NIR-MNCs dispersion were injected into chicken breast tissue. The injection was performed as close as possible to the tissue surface (roughly 1 mm deep). After injection, the tissue was kept at room temperature during 3 h to allow the NIR-MNCs to diffuse into the tissue. After that time, the tissue was placed in a Telesto™ Series SD-OCT Systems from Thorlabs to acquire the optical coherence tomography (OCT) images. This is a pre-configurable OCT Systems that is optimized for high resolution imaging with a 3.5-mm imaging depth while providing a 5.5-µm axial resolution in air. It employs a superluminiscent diode operating at 1300 nm as light source. OCT images were analyzed and treated by using ImageJ software.

Fluorescence NIR-II imaging. Optical excitation was provided by an 808-nm fiber-coupled laser diode. A short-pass filter was used to block the long-wavelength tail of the laser source. The scattered light emitted from the sample was transmitted through a long-pass filter (Thorlabs FL0850), which suppresses the reflected laser signal. Shortwave infrared (SWIR) lenses were then used as a relay to image the exit aperture on a Bragg tunable filter (BTF), which, in turn, would select a specific wavelength, *λ*, of the incoming light. This filtered light was then focused, by a second lens, on an infrared camera (ZephIRTM 1.7) to produce a monochromatic image. Synchronous tuning of the BTF and image acquisition allowed the obtainment of monochromatic fluorescence images corresponding to different emission wavelengths ranging from 900 to 1700 nm. Under those circumstances, a 3D spatial map of spectral variation (*i.e.*, a hyperspectral cube) was built: the first two dimensions provided spatial information and a third dimension accounted for the spectral information. The intensity values of a particular pixel in a hyperspectral cube characterized its spectral fingerprint. For the cases where a broadband NIR-II was desired, the Bragg Tunable Filter was taken out of the optical path followed by the light generated by the luminescence thermometers.

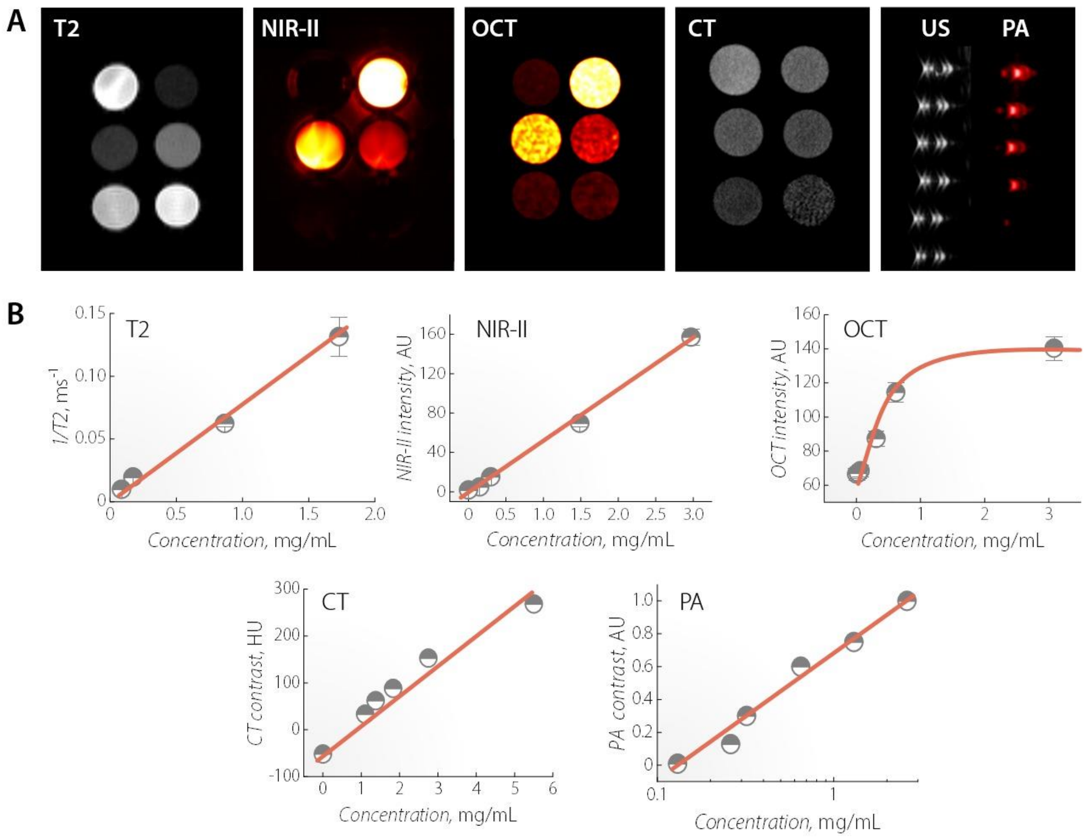

Figure S8. A) MRI, NIR-II, OCT, CT, and PA/Ultrasound images of a series of colloidal dispersions of NIR-MNCs at different concentrations. **B)** Variation of the imaging contrast in each modality as a function of the NIR-MNC concentration. Symbols are data obtained from the corresponding images and solid lines are guides for the eye.

S8. System for *in vivo* magnetic hyperthermia measurements.

AC GEN system is a semi-automate equipment for generation of alternating magnetic fields with an optical access of 20×10 mm to the inner cylindrical part of the coil manufactured by Nanotech Solutions (Spain; **Figure S9**). The value of the field frequency ranges from 50 up to 200 kHz, with a field intensity of up to 20 kA/m throughout the full frequency range. The field intensity varies < 4% along the top-view field region. The surface temperature inside the cylinder can be set thanks to a variable cooling bath for hosting anesthetized small animals along the cylinder length.
The temperature of the magnetic suspension was measured with a commercial optical fibre probe TS2/2 connected to a FOTEMP2-16 two-channel signal conditioner from Optocon AG with an experimental error of ± 0.2 ºC.

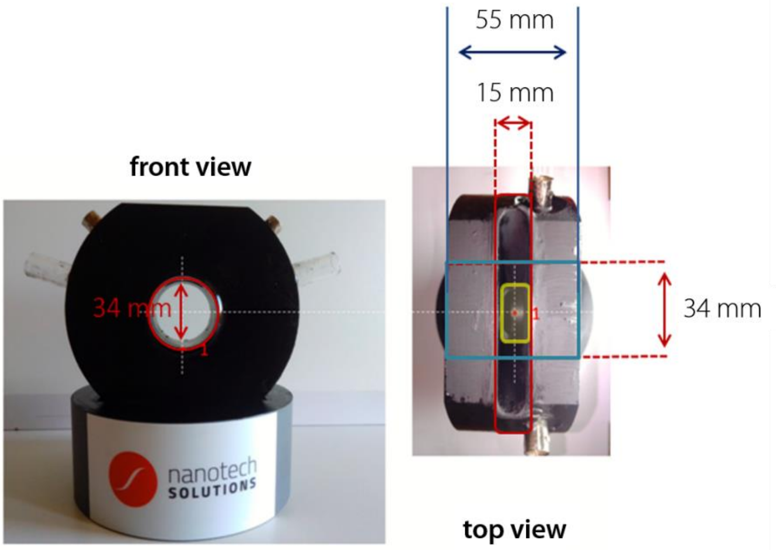

Figure S9. Front and top view of the AC GEN generator. Front view: the red solid circle defines the front cylinder dimension where alternating magnetic field is applied. Top view: Blue solid lines define the inner cylindrical part of the coil. Red solid frame defines the upper region where the alternating magnetic field is applied. The yellow solid rectangle corresponds to the optical window: 10×20 mm.

S9. Calibration of the luminescence thermometer.

In order to test if the nanocapsules were sensitive enough for *in vivo* thermal imaging, an infrared imaging system (IR-VIVO 20219 by Photon Etc) was used to obtain the thermal dependence of their spectral hypercube, *i.e.* a set of luminescence images corresponding to wavelengths found in the 900-1600 nm range. This system was composed of a filter wheel for multispectral filtering, a HyperCubeTM for hyperspectral filtering and an InGaAs camera (ZephIR 1.7TM) for detection (see **Figure S10**). The samples, in contact with a thermoelectrically Peltier plate (SEGA INVEX 2018-0110), were put on a supporting platform at a suitable distance from the InGaAs camera and illuminated by a fibre-coupled diode laser operating at 808 nm (whose distance from the sample was set to 16 cm). A thermocouple was used to monitor the internal temperature of the dispersion. The illumination power density was set to 50 mW/cm^2. The luminescence was then collected and spectrally analysed by the HyperCubeTM (spectral resolution: 6 nm) before getting to the InGaAs camera. The integration time was fixed to 1 s. For the recording of the luminescence images and spectra, the PHySpecTM software, provided by manufacturers, was utilized.

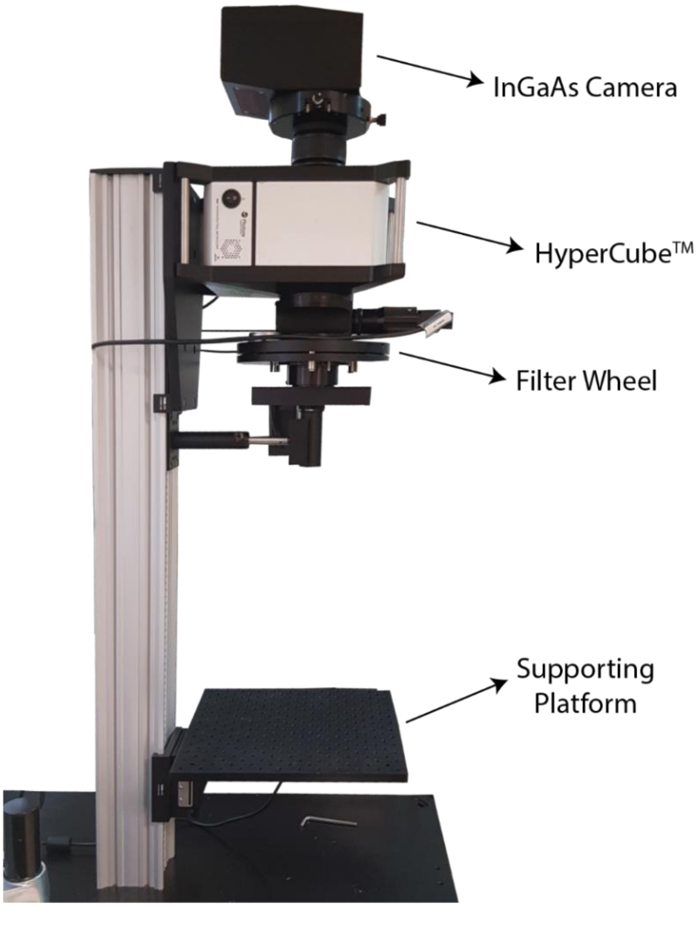

Figure S10. Representative picture of the infrared imaging system used for calibrations measurements.

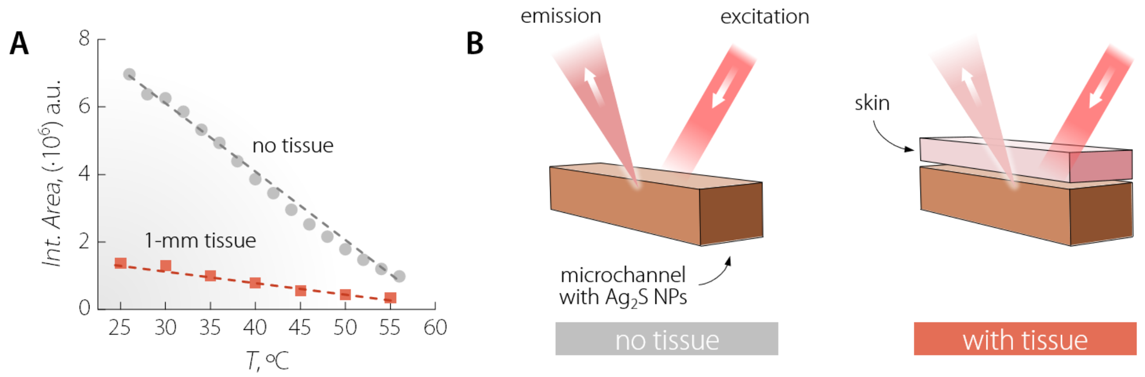

Figure S11. A) Calibration curves for the NIR-MNCs as obtained recording the luminescence signal from an aqueous dispersion in a microchannel in the presence (orange squares) and absence (grey circles) of a 1-mm thick piece of mouse skin. The difference in the integrated intensity of the NIR emission in the two cases is almost one order of magnitude. Although under conditions of slowly varying temperature the sensitivity extracted from the calibration recorded in the absence of tissue could be used to retrieve the temperature *via* an iterative strategy (see details in [16]), the use of the calibration curve obtained in the presence of the tissue allows to better account for any possible interference brought about by tissue-photons interactions. **B)** Graphical representation of the two measurements in the absence and presence of tissue (skin).

S10. *In vivo* magnetic hyperthermia experiments.

Procedures involving animal experiments were approved by the regional authority for animal experimentation of the Comunidad de Madrid and were conducted in agreement with the Universidad Autónoma de Madrid Ethics Committee, in compliance with the European Union directives 63/2010UE and Spanish regulation RD 53/2013.
For this study, a CD1 female mouse (20-22 weeks old) was used. Deep anesthesia was induced with 3% of inhaled isoflurane under a O_2 constant flow rate of 1.5 mL/min, and maintained with 2% of isoflurane and 0.5 mL/min of O_2 during the experiment. The anesthetized mouse was placed on a temperature-controlled plate set to 36 °C, and a thermal probe was used to monitor mouse core temperature. One hundred μL of NIR-MNCs at a concentration of 1.5 mg/mL dispersed in a 0.9% NaCl solution were administered through intradermal injection in the right dorsal area of the mouse back. Following this injection, the NIR-MNCs were located roughly 1.0 mm below the skin's surface. The anesthetized animal was placed in prone position in the experimental setup. An illumination field of around 1 cm^2 was positioned onto the area corresponding to the injection site using a 800 nm fiber-coupled laser diode, and a thermal camera was used to monitor the superficial temperature skin changes throughout the experiment.

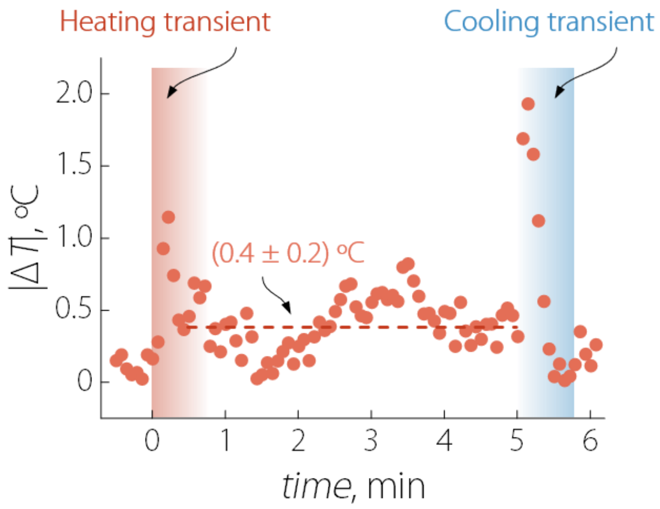

Figure S12. Absolute difference between the thermal readout obtained from the thermal camera and luminescence thermometry as reported in **Figure 4G** of the main text. Since the sampling time for the two approaches is different, to obtain this plot we interpolated the dataset obtained with the thermal camera to the X-values of the dataset obtained from the luminescent thermometers. As expected, during the transients (heating and cooling observed just after turning on and off the AC magnetic field, respectively) the differences are larger, instantaneously reaching almost 2 ºC in the case of the cooling part. However, throughout the magnetic hyperthermia treatment, the fluctuations are of (0.4 ± 0.2) ºC. These differences were expected, due to the different depth probed with the two methods. In these thermal readouts one has also to consider the variations introduced by fluctuations in the power of the excitation source. The presence of a constant mismatch of 0.4 ºC between the temperatures retrieved via the two methods during the hyperthermia treatment is consistent with the difference of the measured locations (i.e., the skin surface *vs* intradermal).

S11. *Ex vivo* experiments with and without tissue.

In order to demonstrate the potential of Ag_2S luminescent nanothermometers to provide reliable sub-tissue thermal measurements, we designed a simple experiment in which a droplet of a suspension of NIR-MNCs was deposited on a microscope slide. The slide containing the NIR-MNCs was then placed inside the magnetic coils and the temperature increment caused by the NIR-MNCs was then measured by both a thermal camera and by analyzing the temporal evolution of the NIR-II emitted intensity generated by Ag_2S NPs. For sub-tissue experiments, a 2-mm thick tissue (chicken breast) was then placed between the microscope slide and the cameras (NIR and thermal). The temperature increment was registered during the application of a 20-kA/m 140-kHz magnetic field for 5 min. **Figure S11** shows the temperature evolution in presence and absence of the tissue as measured by the thermal camera. Without any tissue, upon applying the magnetic field, the thermal camera registered a NIR-MNC-induced temperature increases of 3.5 ºC. In the presence of tissue this temperature increment significantly reduced to 1 ºC. This occurs because the thermal camera reports the temperature at the surface and not the temperature at the NIR-MNC location. Due to heat diffusion, the temperature at the tissue's surface is significantly lower than the temperature of NIR-MNCs. On the other hand, the AC magnetic field-induced temperature increment obtained from the analysis of the NIR-II luminescence in presence and absence of the tissue are virtually identical. It is evident that the use of luminescent thermometry allows real acquisition of sub-tissue temperature as it is based on the analysis of the luminescence generated at the heat source.

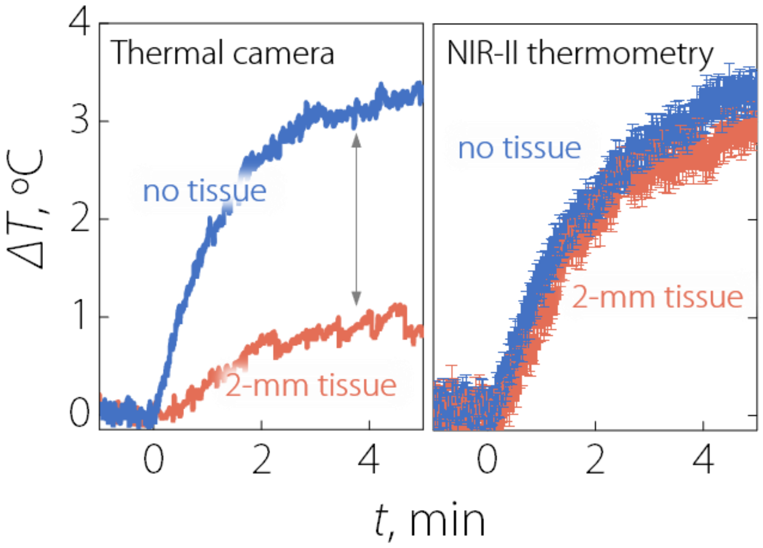

Figure S13. Temporal evolution of the temperature increment induced by the application of an AC magnetic field (20 kA/m, 140 kHz) on NIR-MNCs as measured by a thermal camera and by using the NIR-II fluorescence of Ag_2S NPs in presence and absence of a 2-mm tissue.

S12. References.

1. Ruiz, D., et al., *Synthesis and characterization of Ag_2S and $Ag_2S/Ag_2(S,Se)$ NIR nanocrystals.* Nanoscale, 2019. **11**(18): p. 9194-9200.
2. Sun, S. and H. Zeng, *Size-controlled synthesis of magnetite nanoparticles.* Journal of the American Chemical Society, 2002. **124**(28): p. 8204-8205.
3. Dubertret, B., et al., *In Vivo Imaging of Quantum Dots Encapsulated in Phospholipid Micelles.* Science, 2002. **298**(5599): p. 1759-1762.
4. Al-Jamal, W.T., et al., *Lipid−Quantum Dot Bilayer Vesicles Enhance Tumor Cell Uptake and Retention in Vitro and in Vivo.* ACS Nano, 2008. **2**(3): p. 408-418.
5. Johnson, N.J.J., et al., *Compact Micellization: A Strategy for Ultrahigh T1 Magnetic Resonance Contrast with Gadolinium-Based Nanocrystals.* ACS Nano, 2016. **10**(9): p. 8299-8307.
6. Tong, S., et al., *Self-Assembly of Phospholipid–PEG Coating on Nanoparticles through Dual Solvent Exchange.* Nano Letters, 2011. **11**(9): p. 3720-3726.
7. Russel, W.B., D.A. Saville, and W.R. Schowalter, *Colloidal Dispersions*. Cambridge Monographs on Mechanics. 1989, Cambridge: Cambridge University Press.
8. Faure, B., G. Salazar-Alvarez, and L. Bergström, *Hamaker Constants of Iron Oxide Nanoparticles.* Langmuir, 2011. **27**(14): p. 8659-8664.
9. Liu, Y., et al., *Interpreting the effects of natural organic matter on antimicrobial activity of Ag_2S nanoparticles with soft particle theory.* Water Res, 2018. **145**: p. 12-20.
10. Yang, F., et al., *The ionic strength dependent zeta potential at the surface of hexadecane droplets in water and the corresponding interfacial adsorption of surfactants.* Soft Matter, 2017. **13**(3): p. 638-646.
11. Lin, S. and M.R. Wiesner, *Exact Analytical Expressions for the Potential of Electrical Double Layer Interactions for a Sphere−Plate System.* Langmuir, 2010. **26**(22): p. 16638-16641.
12. Liu, J., et al., *Colloidal Interactions between Asphaltene Surfaces in Aqueous Solutions.* Langmuir, 2006. **22**(4): p. 1485-1492.
13. Messina, R. and Spiteri, L. *On the interaction of dipolar filaments*. Eur. Phys. J. E, 2016. **39** (81): p. 1-9.
14. Auernhammer, G.K. et al. *Viscoelasticity of suspensions of magnetic particles in a polymer: Effect of confinement and external field*. J. Chem. Phys. 2006. **124** (20): p. 204907.
15. K. Wiercigroch-Walkosz, J. Cichos, E. Wysokińska, G. Rotko, W. Kałas, M. Karbowiak, *Near-Infrared Ag_2S quantum dots loaded in phospholipid nanostructures: Physical properties, stability and cytotoxicity* Colloids and Surfaces A: Physicochemical and Engineering Aspects 2019, **579**, 123631.
16. Y. Shen, et al., *In Vivo Spectral Distorsions of Infrared Luminescent Nanothermometers Compromise Their Reliability*, ACS Nano, 2020, **14**(4), 4122-4133.
